# Recognition of Uni-Stroke Characters with Hand Movements in 3D Space Using Convolutional Neural Networks

**DOI:** 10.3390/s22166113

**Published:** 2022-08-16

**Authors:** Won-Du Chang, Akitaka Matsuoka, Kyeong-Taek Kim, Jungpil Shin

**Affiliations:** 1 Department of Artificial Intelligence, Pukyong National University, Busan 48513, Korea; 2 Softbrain Co. Ltd., Tokyo 103-0027, Japan; 3 School of Computer Science and Engineering, The University of Aizu, Fukushima 965-8580, Japan

**Keywords:** character recognition, non-touch character input, human computer interface, gesture recognition, deep neural network, pattern recognition

## Abstract

Hand gestures are a common means of communication in daily life, and many attempts have been made to recognize them automatically. Developing systems and algorithms to recognize hand gestures is expected to enhance the experience of human–computer interfaces, especially when there are difficulties in communicating vocally. A popular system for recognizing hand gestures is the air-writing method, where people write letters in the air by hand. The arm movements are tracked with a smartwatch/band with embedded acceleration and gyro sensors; a computer system then recognizes the written letters. One of the greatest difficulties in developing algorithms for air writing is the diversity of human hand/arm movements, which makes it difficult to build signal templates for air-written characters or network models. This paper proposes a method for recognizing air-written characters using an artificial neural network. We utilized uni-stroke-designed characters and presented a network model with inception modules and an ensemble structure. The proposed method was successfully evaluated using the data of air-written characters (Arabic numbers and English alphabets) from 18 people with 91.06% accuracy, which reduced the error rate of recent studies by approximately half.

## 1. Introduction

Hand gestures along with speech are important tools in human communication. Notably, hand gestures have allowed people to express and describe their thoughts more accurately. In addition, gestures are more compelling in articulating one’s opinions and beliefs, especially when there are difficulties in voice communication, the environment is noisy, or the communication occurs in an environment where silence is required.

In recent decades, studies have used gestures as communication tools between humans and computers. Air writing, where researchers write in the air with hand gestures, is recorded, analyzed, and recognized automatically using computer algorithms. The most popular method for recording hand gestures is the camera-based approach using a pen and an optical camera. Detecting a pen-tip or fingertip is one of the most important tasks in such computer vision-based approaches, as the shape of the written characters is very similar to conventional handwriting on paper. Handwriting recognition on paper is a traditional research topic that has been studied in the last decades. Various methods, such as linear discriminant analysis, multi-layer perceptron, tangent distance, and support vector machine [1,2], have been previously proposed, and the accuracies of handwritten character recognition using convolutional neural networks are higher than 99% in recent papers [3,4].

In [5], the pen tip position was easily obtained with color information, as the color of the pen was fixed in the experiments. In [6], a method to detect hand regions by predefining the color of hands was demonstrated, which requires a controlled environment. Hsieh et al. proposed a method to detect hand trajectory by obtaining the difference between consequent video frames [7].

The detection of the hand or fingertips can be enhanced by substituting the optical camera with a depth sensor. For example, [8] used the software development kit (SDK) of a Kinect sensor to detect the hand position, and [9] calculated the position of the fingertip from the hand position and depth information. In [10], the position of the fingertips was traced, and tapping gestures were recognized using the Kinect sensor to detect the fingertips. Similarly, a hand-tracking method by measuring depth with an ultrasonic transceiver was proposed in [11].

A drawback of the camera- or the depth sensor-based method is that they mostly require users to stand or sit in front of a camera or a sensor. Though head-mounted cameras, proposed in [6], mitigate this limitation to a certain extent, the inconvenience persists.

Another approach to recording hand gestures is using accelerometer/gyro sensors, which provide users freedom of movement. Because accelerometer/gyro sensors are embedded in most present-day smartwatches/bands, users can easily utilize them as input devices. This approach is also stable irrespective of the light conditions.

One of the biggest issues with the accelerometer/gyro sensor-based approach is tracing the position of the sensors in the global *xyz* coordinates. Because the sensors move or rotate while obtaining the signals, the accelerometer values follow the local coordinates of the sensors. Therefore, [12] proposed a method for the automatic conversion of coordinates; however, it requires a ground truth of the traces, which is not commonly available.

Therefore, the accelerometer/gyro sensor-based methods have lower accuracy than camera-based methods. For example, the maximum accuracies of camera-based approaches are 99.571% for Arabic numbers and 16 directional symbols [7], and 95% for English letters and Arabic numbers [8]. By contrast, the accelerometer/gyro sensor-based methods achieved 79.6% accuracy for Japanese Katakana (46 letters) with the K-nearest neighbor method [13], 88.1% for Japanese Hiragana (46 letters) with the hidden Markov model (HMM) [14], and 89.2% by combining K-NN with dynamic time warping (DTW) techniques in [15].

Another issue with the sensor-based approach is the diversity of the signals obtained among individuals. Because the speed and rotating angles of a hand differ for each person, it is difficult to recognize the signal of an unknown person. For example, recognition accuracy dropped significantly from 89.2% to 83.2% in [15] and from 95.3% to 81.9% in [16] when the system was tested independent of the writer.

This study focused on increasing the accuracy of the air-writing method for this writer-independent case. We proposed a method to process the obtained signal by designing a novel network structure, training method, and boosting algorithm for the trained network model.

The remainder of this paper is organized as follows: Section 2 explains the experimental data and proposed method. The experimental results and related discussions are presented in Section 3. Finally, Section 4 concludes the paper.

## 2. Materials and Methods

### 2.1. Device

**Figure 1:**
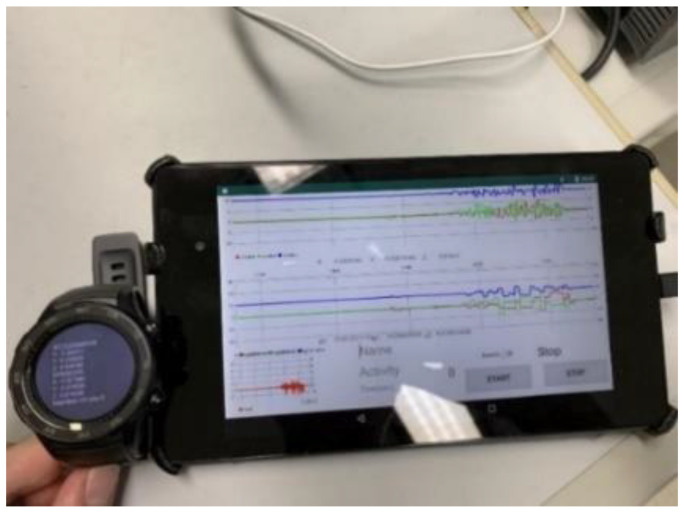
Huawei Watch 2 and tablet. The waveforms transferred from the device are displayed and recorded on the tablet.

A Huawei Watch 2 and an Android tablet (Nexus 7) were used to collect the data. The watch has a built-in 3-axis accelerometer and gyro sensors and can transfer the data into the Android tablet using Bluetooth (see Figure 1).

**Figure 2:**
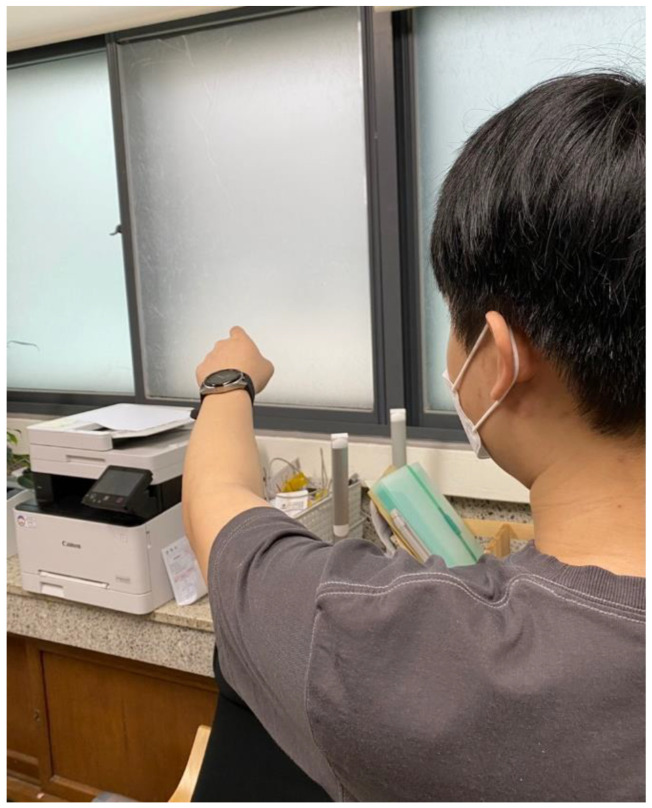
Example of an air-writing activity.

The data were recorded on the tablet as there was insufficient space to record data in the watch. A user wears the watch on a wrist and virtually draws characters with hand movements (see Figure 2).

### 2.2. Data

Twenty people aged from 19 to 24 participated in the air-writing activity. Each person virtually wrote the characters freely in the air five times each, after having the shape of each character explained to them. There was no guidance on arm posture or speed, and the participants were allowed to rest freely during the experiments. Data from the smartwatch were transferred to the tablet and recorded. Data from two participants were excluded from further evaluation because of recording errors found after the experiments. All participants were right-handed.

**Figure 3:**
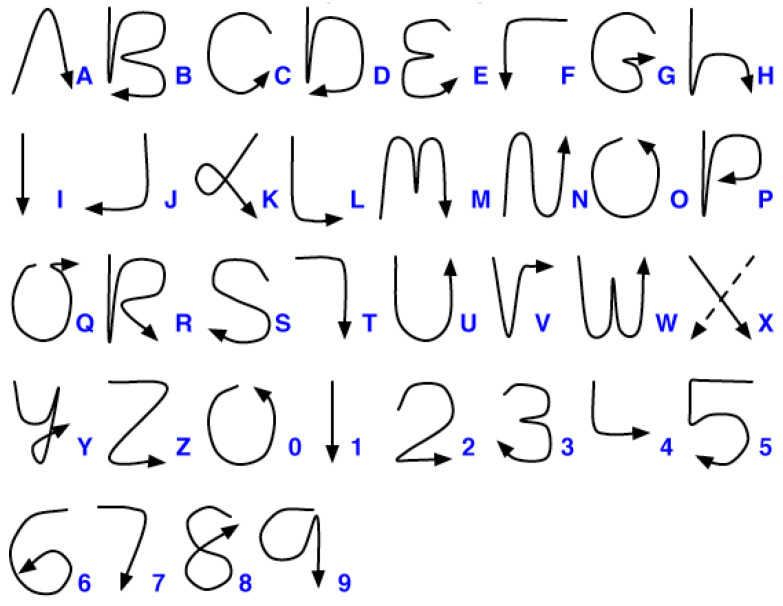
Uni-stroke characters (Graffiti in Palm OS). Reprinted with permission from [17].

Figure 3 shows the Graffiti design of the characters used in this experiment. This was developed for Palm OS-based PDA [17] and was used in the air-writing system in [8]. Because the air-written characters are not visible, the uni-stroke design makes it convenient for users to move their hands to write characters.

**Figure 4:**
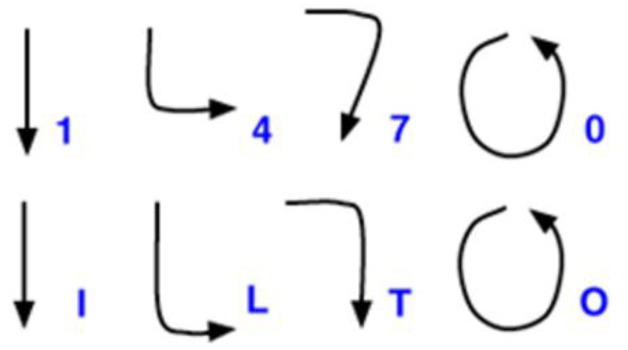
The pairs of the same or slightly different gestures. Reprinted with permission from [17].

The gestures of some characters are the same or slightly different (see Figure 4). The gestures of Arabic numerals “0” and “1” are the same as the gestures of letters “O” and “I,” respectively; the gestures of “4” and “7” are very close to that of “L” and “T,” respectively. Each of the 36 characters were recorded five times by each participant, but the patterns with similar shapes were categorized as a single class during the pattern recognition phase. Therefore, the number of classes of gestures used in the experiment was 32.

The data were transferred and recorded at 40 Hz from the watch to the tablet. The data include accelerometer and gyro values in three axes and the gravity-removed accelerometer values provided by the watch using a linear filter.

**Figure 5:**
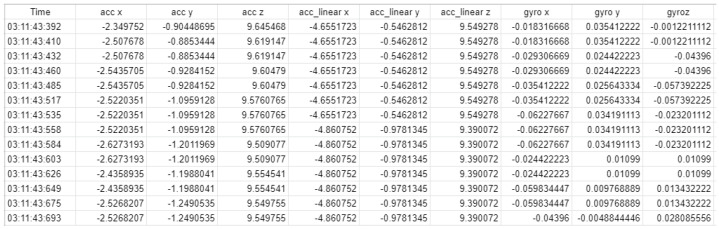
Example of collected data (digit 0) from a smartwatch.

An example of the collected data in a tabular form is shown in Figure 5. The first column has the time stamp, the 2nd to 4th columns have the acceleration data, the 5th to 7th columns have acceleration data after gravity removal, and the 8th to 10th columns have the gyro data.

**Figure 6:**
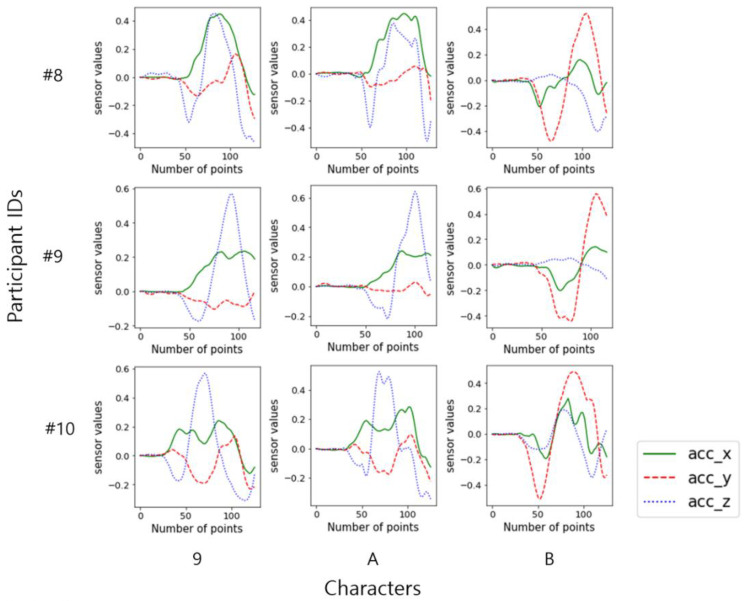
Acceleration signals of three characters by three participants.

Figure 6 shows examples of a character as graphs written by different writers. The trends in the signals of the same gestures are similar, but there is diversity among writers.

### 2.3. Preprocessing

The collected data were smoothed using a Savitzky–Golay filter [18]; this was conducted as the precision of the raw data was low because of quantization. The window length and polynomial order were set to 13 and 5, respectively. This also removed high-frequency noise. However, some of the signals were corrupted during the recording phase. Therefore, signals shorter than the median length of the same gesture by each participant were removed from the dataset. A total of 18 out of 3240 signals were removed from 18 participants’ data. The removed signals were distributed over 12 characters, where the maximum number of the removed signals per character was 4 of Arabic digit 3.

**Figure 7:**
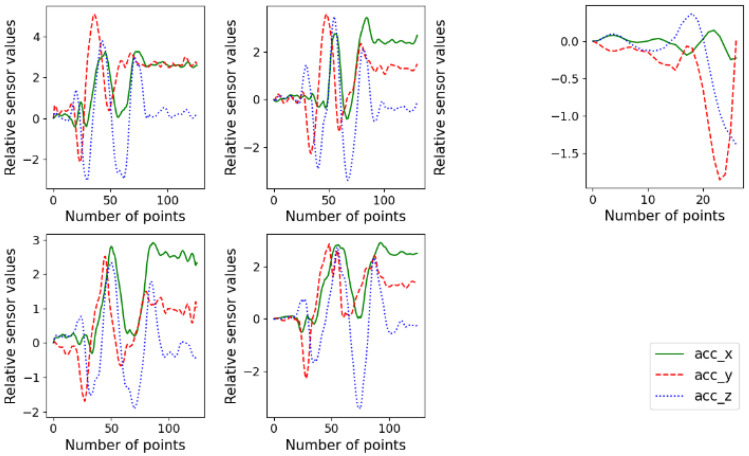
Example of normal (**left**) and corrupted (**right**) signals of Arabic numeral 9 written by a participant.

Examples of normal and corrupted signals of the accelerometer are shown in Figure 7. The accelerometer values were adjusted (shifted along the *y* axis) such that the value at the beginning is zero.

Because the obtained signals include the non-movement data, the following algorithm was developed to trim the signals using wavelet transforms:

(1) Calculate detail coefficients from single-level wavelet transform for each channel of signals;
(2) Interpolate and smooth with a Savitzky–Golay filter [18] as the length of the coefficient signal is half of the original signal;
(3) Calculate the standard deviation of the signal for each channel, and set the minimum value among the channels as the threshold for the data;
(4) Signal regions where the detail coefficients of all the channels are less than or equal to the threshold are marked as non-movement regions;
(5) Remove the signals in the non-movement regions.

**Figure 8:**
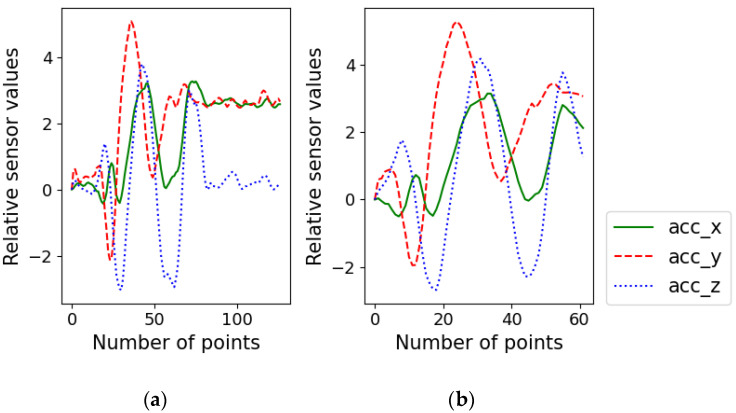
Example of non-movement region removal for Arabic numeral 9. (**a**) Accelerometer signal before the removal, (**b**) signal after the removal.

This algorithm is applied for each datum separately. Removing the non-movement region means that the signal changes in this region are ignored and the data points are removed. Figure 8 shows an example of non-movement region removal. The regions at the beginning and end were removed, and only the hand movement regions were retained.

As the lengths of the processed signal varied, all signals were resampled to have the same number of data points. This process was necessary to input the data into a convolutional layer. The resampled signal length was 129, which is the maximum length of the hand movement regions for all the data.

The data were normalized between 0 and 1, which is the recommended input for artificial neural networks. Normalization was conducted for each channel group of accelerometers, linear accelerometers (gravity-removed accelerometers), and gyro values. The minimum and maximum values were calculated for each group and then normalized to between 0 and 1. The minimum and maximum values were then calculated for each air-writing datum.

### 2.4. Convolutional Neural Network

**Figure 9:**
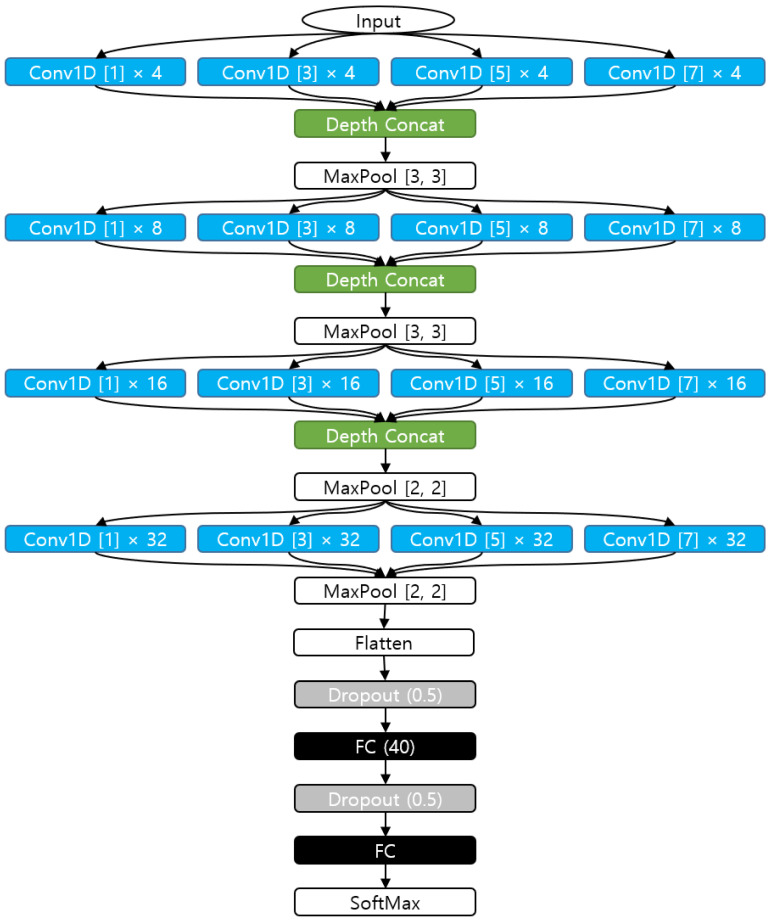
Proposed network structure. Conv1D[N].

The proposed network model with inception architecture [19], inspired by GoogLeNet [20], is shown in Figure 9. The two-dimensional (2D) convolutions are substituted by 1D convolutions and the inception modules of GoogLeNet were simplified for our purposes. The inception modules are expected to extract features in different frequency levels. Different sizes of convolutions were located parallelly and the results were concatenated into a tensor after the convolutions. The sizes of the convolution filters are 1, 3, 5 and 7. The number of filters is 4 for the first inception module, and the numbers of the succeeding modules are 8, 16, 32 and 64.

Fully connected layers were connected with the dropout layers to prevent overfitting, as the amount of data is limited in our experiment. Parameters such as the number of filters, filter sizes, and dropout ratios are shown in Figure 9.

**Figure 10:**
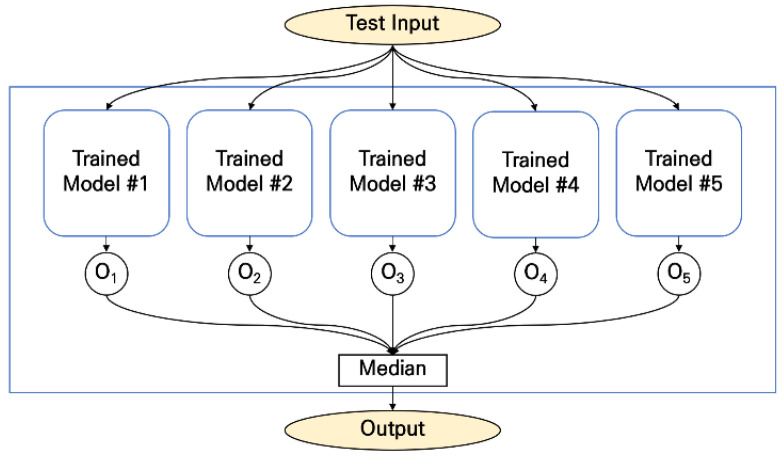
Ensemble method.

The accuracy of artificial neural networks may vary because of the randomness of the training algorithms, especially when the size of data is small. We employed an ensemble model to stabilize and increase the overall accuracy [21,22]. We trained five different models in the training phase and calculated the median values from the five outputs to derive the final output (see Figure 10).

The proposed method was validated independently for writers by training the network with the data of 17 participants and then testing using the data of the last participant. This process was repeated 18 times such that each participant’s data were used as test data at least once.

The number of epochs and batch size were set to 400 and 16, respectively. The ADAM optimizer with a learning rate of 0.001 and the AMSGrad option [23] was used for training the network. Weights were initialized with Xavier method. These parameters and options were determined experimentally.

### 2.5. User-Independent Evaluation

The proposed method was evaluated in a user-independent manner, following the leave-one-subject-out cross-validation (LOSOCV) approach [24]. In this approach, data of a participant were used for testing, and the other data were used for training (20% of the training data were selected for validation in the training phase). The training and test were repeated 18 times, such that all the data were used for the test. The biggest merit of LOSOCV is that it enables user-independent evaluations. Because the data from the same participant were separated, it can be assumed that the accuracies with a new user’s data would be similar to the results of the evaluation [25].

## 3. Results

The experiment was conducted on a computer with Intel i9 and Nvidia RTX 3090ti. The GPU memory was 24GB and all the program codes were written in Python with the TensorFlow library.

The proposed method was evaluated with and without the preprocessing trimming algorithm, as it is often reported that CNNs show improved performance without intensive preprocessing algorithms. The accuracy with and without the trimming procedure was 88.27% and 91.06%, respectively, indicating the ineffectiveness of the trimming algorithm in our dataset. This may have been caused by writing speed changes, as removing the non-movement regions was successful in our visual examinations. The convolutional operators of the networks could fail to detect signal changes when the writing speed changes when adjusting the length of the signals after trimming.

The ensemble structure was effective and stable according to the experiment. The accuracy dropped to 89.51% when the ensemble structure was removed from the proposed method and did not improve even with repeated experiments. This is because we utilized the 18-fold validation policy where the accuracies were averaged from 18 different trained models.

Table 1 compares the results of the proposed method and the conventional methods in the literature. This indicates that the proposed method increased the accuracy by utilizing the uni-stroke design and a deep neural network, reducing error rates by approximately half, from 16.8% as reported in [15] to 8.94%. It was reported that the error rate was reduced to 4.4% with a word-level correction algorithm in [15], but this was not reported in Table 1 to compare the accuracies of a character-level recognition.

It is remarkable that the proposed method achieved accuracy higher than 90% with data from accelerometer and gyro sensors in a user-independent manner. Higher accuracies have been reported with depth sensors or optical cameras, but the best accuracies with accelerometer and gyro sensors have been limited to 83.2% when the systems were validated user-independently. The high accuracy was achieved by two factors: the dataset and network structure. We employed the uni-stroke characters designed for PDA, which are simpler than the original alphabets. Because the uni-stroke characters were designed for finger gestures, we expect that it was easier for the participants to be accustomed to air writing. The design of the proposed network structure also affected the accuracy. A simplified inception structure and the sizes and numbers of filters affect the accuracy of the neural networks.

The evaluation was conducted in a user-independent manner to overcome the limitations of the small size of the dataset. The same accuracies are expected to be achieved when new users’ data are included for additional evaluations. Because of the posture variations during the air writing, the accuracies of the user-independent validations were lower than the user-dependent validations [15,16].

**Figure 11:**
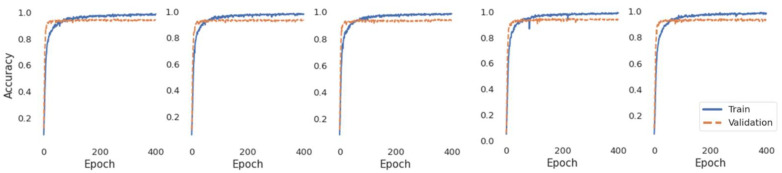
Example of training and validation curves when a model is trained five times.

Ninety models were trained during the experiment as the five trained models were utilized for each fold. Figure 11 shows the training and validation curves when we trained the same structure of the proposed model five times. The curves of the five models had similar trends. The accuracies of the training and validation increased sharply during the first 100 epochs and were stable after the 200th epoch.

The five models were trained with the same network structure and data, but the predicted outputs were different because of the randomness of the weight initializations. Accuracy without the ensemble algorithm was slightly reduced to 89.51% on average.

Table 2 shows the accuracy changes according to the number of epochs. Accuracy increased from 89.48% to 91.06% as the number of epochs increased from 50 to 400, but it gradually decreased after 400 epochs.

The differences in the accuracies were observed for each character. Table 3 shows the precision, recall, and F1 scores for each character. The means of F1 score, precision, and recall were 90.91%, 91.09% and 90.81%, respectively. Some characters were considered as being in the same categories because they have little difference in gesture (0–O, 1–I, 4–L, and 7–T). Some characters showed relatively lower scores (with a standard deviation of 5.07). Only a single character had an accuracy under 80% (“Z” was 79.78%).

**Figure 12:**
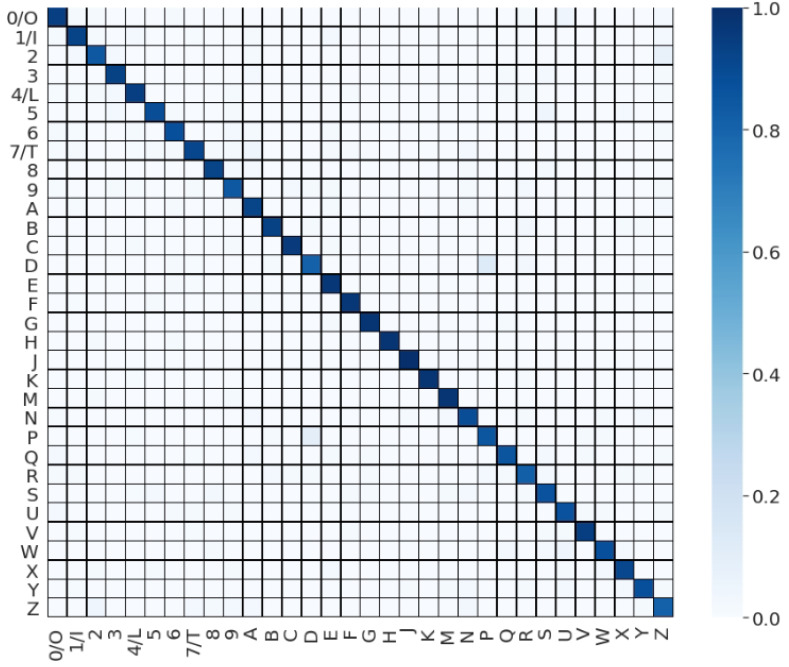
Confusion matrix.

One of the reasons for this low accuracy can be found in the confusion matrix in Figure 12. The main confusion characters are Z–2 (four errors) and Z–7/T (four errors). These errors can be explained by the similar shapes of these character pairs in the 3D acceleration space. For example, D and P have similar acceleration changes, except for the power along the vertical axis when drawing an arc at the end.

**Figure 13:**
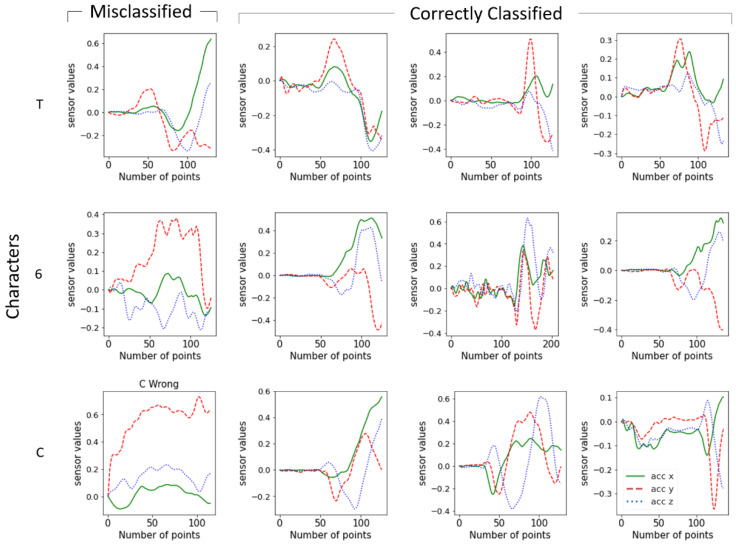
Examples of misclassified and correctly classified signals of characters.

Figure 13 shows the misclassified signals of characters together with the correctly classified signals. The misclassified characters generally showed different signal trends. For example, misclassified T and 6 had additional movements in the Y-axis in comparison to the correctly classified characters. This can be caused by unexpected wrist rotation during the air writing or a loosened strap meaning the watch moved freely during the air writing. Some signals were completely different signal shapes as shown in the signals of C. It is expected that the participant may have moved their arm in the wrong direction during the air writing.

**Figure 14:**
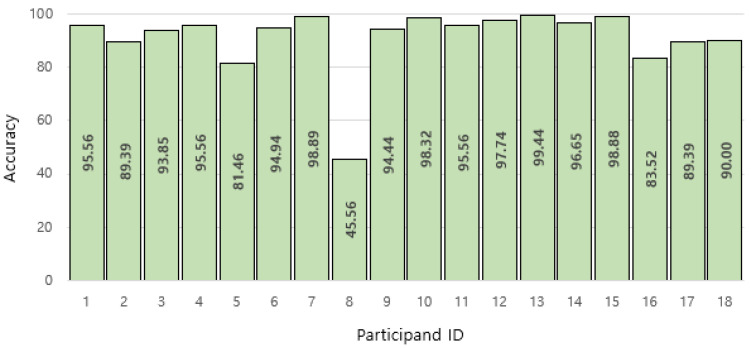
Accuracies according to the participants.

Accuracies also varied according to the participants (see Figure 14). The accuracies of the 13 participants were higher than 90%; four participants had accuracies between 80% and 90%, and one (participant #8) had 45.56%, which is approximately half of the average. The reason for this low accuracy is possibly a different method of hand/arm movements during air writing. Accuracy may increase with additional training data for various postures.

It should be noted that the data of two participants were excluded from the experiments because of a recording failure, since similar situations could occur during real-time application in the future. Firstly, this recording issue could be recognized by checking the data communication status, as the data are corrupted by network failure. Secondly, recording failure can be recognized by checking the length of the obtained data. Because recording failure can occur for unknown reasons, we need to check if all the data were transferred by checking the length of the obtained signals.

## 4. Conclusions

This paper proposed a method for recognizing the air-written characters of English alphabets and Arabic numbers from accelerometer and gyro sensor data. Accuracies of air writing with accelerometer and gyro sensor data have been lower than depth sensors or optical cameras because of data variations among users. This study utilized a uni-stroke design for the characters and a deep neural network structure to solve this problem. The network structure was newly designed by utilizing simplified inception modules. The method was evaluated using user-independent data from 18 people to validate whether the proposed method overcomes the variation problems among users. The proposed user-independent air-writing system achieved 91.06% accuracy, which is higher than what has been reported in previous studies using acetometer/gyro sensor data, reducing the error rates by approximately half.

One of the limitations of the proposed system is the variation in accuracies according to the characters and the participants. Designing new shapes for English alphabets could be a solution because much confusion occurred between characters with similar shapes in the air writing. Combining boosting algorithms such as AdaBoost with the convolutional neural network [27] could be another solution. The use of a larger database for the network training would reduce the error rates for unknown styles of air writing.

## Figures and Tables

**Table 1:** Comparison of the studies on air-writing hand gesture recognition.

Reference	Recording Devices	Algorithms	Patterns	Number of Participants	Accuracy
[8]	Kinect	DP matching	Alphanumeric characters	10	95.0% (Alphanumeric character)
					98.9% (Arabic digits)
[9]	Kinect	Benchmarks	Arabic digits, alphabets, and symbols	8	96.5%
[10]	Kinect	Template matching	Japanese (Hiragana) and alphabets	-	94.3%
[7]	Optical camera	Convolutional neural network (CNN)	Arabic digits and 16 directional symbols	14	99.571% (writer-independent)
[5]	Optical camera	CNN	Arabic digits	20	97.7%
[11]	Ultrasonic transceiver	CNN with long short-term memory (LSTM)	Arabic digits (1 to 4) and alphabets (A to D)	-	98.28%
[6]	Wearable optical/infrared camera	DP matching	Alphanumeric characters	5	75.5%
[26]	Smartwatch	Naive Bayes	Alphabets	1	90.00% (Naive Bayes)
		Logistic regression			94.62% (logistic regression)
		decision tree			88.08% (decision tree)
[16]	Wearable motion sensors	Hidden Markov model (HMM)	Alphabets	10	95.3% (writer-dependent)
					81.9% (writer-independent)
[15]	Smart bands	K-nearest neighbor (k-NN) with dynamic-time-warping (DTW)	Alphabets	55	89.2% (writer-dependent, k-NN + DTW)
		CNN			83.2% (writer-independent, CNN, character level)
[14]	Wii Remote Controller	Hidden Markov model (HMM)	Japanese (Hiragana)	5	88.1%
[13]	Laser pointer with accelerometer	K-NN	Japanese (Katakana)	10	79.6%
-	Proposed	CNN with ensemble structure	Alphanumeric characters	18	91.06% (writer-independent)

**Table 2:** Accuracies with different numbers of epochs.

Epochs	50	100	150	200	300	400	500	600
Accuracy	89.48	89.66	90.41	90.35	90.75	91.06	90.78	90.29

**Table 3:** Accuracies according to the characters.

Character	Precision	Recall	F1 Score
0/O	89.89	93.89	91.85
1/I	91.26	92.78	92.01
2	88.37	84.45	86.36
3	92.05	93.10	92.57
4/L	92.86	94.41	93.63
5	94.12	88.89	91.43
6	90.80	87.78	89.27
7/T	91.62	91.11	91.36
8	93.26	92.22	92.74
9	84.09	84.09	84.09
A	82.18	92.22	86.91
B	95.40	93.26	94.32
C	96.62	95.56	96.09
D	85.71	80.90	83.24
E	88.54	96.59	92.39
F	95.60	96.67	96.13
G	96.67	97.75	97.20
H	100.00	97.75	98.86
J	97.83	100.00	98.90
K	100.00	97.75	98.86
M	95.65	97.78	96.70
N	86.96	88.89	87.91
P	85.23	85.23	85.23
Q	92.77	85.56	89.02
R	83.91	82.02	82.95
S	83.87	86.67	85.25
U	86.67	86.67	86.67
V	96.59	94.44	95.51
W	94.05	87.78	90.80
X	91.01	91.01	91.01
Y	92.86	87.64	90.17
Z	78.49	81.11	79.78
Avg.	91.09	90.81	90.91

## Data Availability

Not applicable.
